# Self-Adaptive Intelligent Metasurface Cloak System with Integrated Sensing Units

**DOI:** 10.3390/ma17194863

**Published:** 2024-10-02

**Authors:** Panyi Li, Jiwei Zhao, Caofei Luo, Zhicheng Pei, Hui Jin, Yitian Huang, Wei Zhou, Bin Zheng

**Affiliations:** 1 State Key Laboratory of Extreme Photonics and Instrumentation, ZJU-Hangzhou Global Scientific and Technological Innovation Center, Zhejiang University, Hangzhou 310027, China; 22231008@zju.edu.cn (P.L.); hyt20001014@163.com (Y.H.);; 2 International Joint Innovation Center, The Electromagnetics Academy at Zhejiang University, Zhejiang University, Haining 314400, China; 3 Key Laboratory of Advanced Micro/Nano Electronic Devices & Smart Systems of Zhejiang, Jinhua Institute of Zhejiang University, Zhejiang University, Jinhua 321099, China; 4 China E-Tech (Ningbo) Maritime Electyonics Research Institute Co., Ltd., Ningbo 315000, China; 5 No. 36, Institute of CETC, Jiaxing 314001, China

**Keywords:** adaptive metasurface cloak system, direction recognition, integrated sensing units, phase interferometry

## Abstract

Metasurfaces, which are ultrathin planar metamaterials arranged in certain global sequences, interact uniquely with the surrounding light field and exhibit unusual effects of light modulation. Many interesting applications have been discovered based on metasurfaces, particularly in invisibility cloaks. However, most invisibility cloaks are limited to working in specific directions. Achieving effectiveness in multiple directions requires the metasurface to be designed with both perception and modulation capabilities. Current multi-directional metasurface cloak systems are implemented with discrete components rather than an integrated sensing component. Here, we propose an intelligent metasurface cloak system that integrates sensing units, resulting in the cloaking effect with the help of a real-time direction sensor and an adaptive feedback control system. A reconfigurable reflective meta-atom based on phase modulation is presented. Sensing units replace parts of the meta-atoms in the designed tunable metasurface, integrating with an FPGA responsible for measuring the direction and frequency of the incident wave, constituting a closed-loop system together with the feedback parts. Experimental results demonstrate that the metasurface cloak system can recognize the different directions of the incoming wave, and can adaptively manipulate the reflected phase of EM waves to conceal objects without any human participation.

## 1. Introduction

Metamaterials are a new type of artificial composite materials composed of sub-wavelength artificial atoms arranged in a periodic or regular aperiodic manner. Metasurfaces can be regarded as two-dimensional planar forms of metamaterials. By changing the geometric structure and arrangement of units, the electromagnetic (EM) parameter can be regulated, leading to specific EM properties of metasurface [1,2,3,4,5,6,7]. Based on this, metasurfaces have been widely applied in the field of optics, such as vortex beam shaping [8,9], optical holography [10,11], focusing [12,13], and energy harvesting [14,15], while also providing new design ideas for the recently highly anticipated invisibility cloaking. For example, by utilizing a phase-modulation metasurface, the reflection phase of a bump can be adjusted to restore the phase of the reflected beams, as if the wave had occurred on a plane mirror [16,17]. The ultrathin invisibility skin cloak based on phase modulation addresses the problem of bulkiness compared to other invisibility cloaks. It has sparked plentiful research interests and encouraged new inventions, such as the dual-wavelength carpet cloak [18] and the ultrathin terahertz unidirectional carpet cloak [19].

With the increasingly complex EM environment, higher demands have been put forward regarding the performance of the invisibility cloak. It is hoped that the invisibility cloak is no longer limited to a fixed-wave mode or background environment. On the one hand, this requires the metasurface to be designed with modulation capabilities, as passive metasurfaces [18] develop into active metasurfaces. By integrating discrete active elements in the metasurface, such as varactors [20,21,22,23,24,25], PIN diodes [26,27,28], and MEMS switches [29,30], the EM properties of the metasurface can be modulated to realize different EM functions, including cloaking. A multifunctional polarization-insensitive metasurface cloak, which symmetrically incorporates four varactors, was proposed to solve the stealth problem in the everchanging environment [31]. Coping with different incident waves flexibly and quickly not only requires tunability but also sensing functionality. Intelligent metasurfaces have emerged. By obtaining the information of the incident wave and timely autonomously transmitting it to the control module of the metasurface cloak, a closed-loop system is able to cloak without any manual control or intervention, liberating manpower. An intelligent cloak driven by deep learning was developed by using two detectors, detecting incident angle and the reflected spectrum [32]. An omnidirectional flying cloak has been presented; the gyroscope and camera help in detecting the message of the drone and the surrounding environment [33]. However, most of these existing autonomous invisibility cloaks use additional detection devices, which cannot guarantee that they are in the same position as the cloak, making it difficult to receive completely consistent environmental information. On the other hand, the extra devices are inconvenient and limited in practical applications.

In this paper, we design an intelligent metasurface cloak system that integrates sensing units to adapt to varying EM environments. By applying different direct-current bias voltages across varactor diodes at working frequencies, the metasurface cloak adjusts the local unit phase to restore the reflection phase, thereby achieving cloaking. The sensing unit replaces some of the meta-atoms to receive the incident signal, ensuring full compatibility with the metasurface. A phase interferometer algorithm is employed to estimate the direction of the received wave, ensuring that the sensing unit does not interfere with the metasurface cloak’s phase regulation. All bias voltage is automatically calculated by an adaptive feedback control module and instantly supplied to the cloak. Our work advances cloaking strategies by accommodating changing incident wave modes, and the integration of sensing components enhances the system’s precision and reduces its weight. Simulations and experiments demonstrate the effectiveness of the detection module and the system’s robust capability for adaptive reflection phase adjustment, enabling the cloak to provide effective invisibility for EM waves from various incident directions without human intervention.

## 2. The System’s Design

### 2.1. Architecture of the Intelligent Metasurface Cloak System

**Figure 1:**
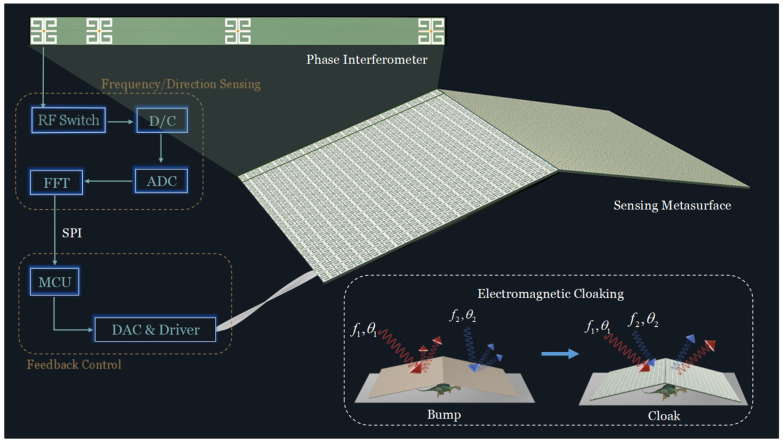
A schematic of an intelligent metasurface cloak system for self-adaptively responding to different directions. It consists of a programmable reflective metasurface with independent phase control, a real-time radio frequency sensing system and an adaptive-feedback electronic control system, constituting a closed-loop system. Integrated sensing units are designed to replace part of the meta-atoms based on a phase interferometer to improve the accuracy and convenience of direction finding. The intelligent metasurface system is able to adaptively detect direction and convert it to voltage corresponding to the compensated phase, restoring the phase of light for full polarization, as if the light was emitted on a flat mirror.

Generally, cloaking by phase modulation is based on specific directional conditions. When the direction changes, the phase to be compensated needs to be recalculated [16,17]. To realize the performance for the invisibility cloak under the premise of no active control and human intervention, the cloaking system must have both perception and regulation capabilities. Currently, the available adjustable invisibility cloak has been designated for the addition of further detector components [32,33], which has the effect of increasing the load on the system and introducing errors. So, we propose the integration of sensing units as the solution. The architecture of the intelligent metasurface cloak system is plotted in Figure 1. It can be roughly divided into the following three parts: the tunable metasurface cloak, the frequency/direction sensing module, and the adaptive feedback control module. This involves designing a metasurface by replacing part of meta-atoms with sensing units. The sensing units integrating with the direction detection module are tasked with receiving and measuring the frequencies and directions of incident EM waves. Through SPI communication, the processed information is transmitted to the feedback control module. The control module then calculates all bias voltages and sends them to the metasurface automatically. Consequently, upon the identification of incident waves in different directions, the system is capable of adapting the phase to cloak in an adaptive manner. In order to implement the corresponding function of feedback regulation, a varactor diode is embedded in the meta-atom. The adjustment of the bias voltage means a change to the value of the varactor, which in turn affects the phase response of each active meta-atom, thereby achieving cloaking.

### 2.2. Reconfigurable Reflective Meta-Atom and Adaptive Metasurface

**Figure 2:**
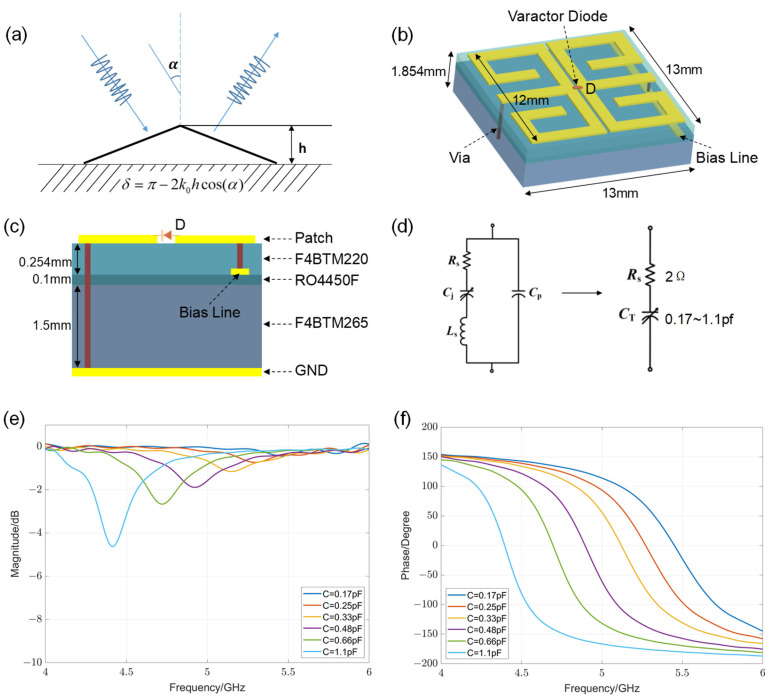
Designed reconfigurable meta-atom with independent phase response. (**a**) A schematic view of the phase modulation invisibility cloak. When light impinges onto the PEC bump, the phase of the reflected light will be distorted. After covering it with an ultrathin metasurface, which can provide an additional phase shift to control the local reflection phase, the previously distorted reflected light will be recovered with the same phase, as if the light impinged onto a flat mirror. (**b**) A three-dimension illustration of the meta-atom (unit cell) with a varactor. (**c**) The topological structure of the proposed meta-atom unit. (**d**) The SPICE model of the MA46H120 varactor diode and the equivalent circuit model used to represent the varactor diode in the full-wave simulation. (**e**) Simulated reflection amplitude curves of the meta-atom for different capacitance under y-polarized incidence at the frequency range of 4–6 GHz. (**f**) Simulated reflection phase response curves for different capacitance under y-polarized incidence at the frequency of 4–6 GHz.

In order for the metasurface to reflect scattered waves similar to those produced by the metal plate without hidden objects, for an oblique-angle light incident on a bump at a height of h with respect to the reference plane (Figure 2a), the introduced phase shift should compensate for the phase difference between the light scattered by the object and that reflected from the reference plane [16]. The formula for calculating phase difference is $\delta =\pi -2k_{0}h\cos(\alpha )$, where $k_{0}$ is the free space wave-number. Thus, it is essential to have the phase shifts covering nearly 0 to 2π for the designed meta-atom. Figure 2b shows our designed unit with a complex metal pattern and embedded varactor diode. The entire structure has two layers of dielectric substrates, as shown in Figure 2c. The metal patterns are etched on the top layer of the F4BTM220 dielectric substrate with a relative permittivity of 2.2 and a thickness of 0.254 mm. The lower layer is the F4BTM265 dielectric substrate with a relative permittivity of 2.65 and a thickness of 1.5 mm. An RO4450F film with a relative permittivity of 3.52 and a thickness of 0.1 mm bonds the above two kinds of dielectric substrates. At the bottom of all the layers is a full metal sheet. The microwave varactor diodes are connected between two metallic patterns symmetrically placed on the top of the dielectric substrate. One pole of the varactor is grounded through a metalized via hole, and the other one is connected to a DC bias line etched on the bottom of the F4BTM220 dielectric substrate. In this work, the MA46H120 commercial varactor manufactured by MA-COM Technology Solutions Company is chosen, whose SPICE model is shown in Figure 2d. In the simulation, it can be equivalent to a resistor Rs and a capacitor Ct connected in series for simplicity, where the resistance value is fixed at 2 Ω and the capacitance value can be adjusted between 0.17 and 1.1 pf.

The EM performance of the designed meta-atom is simulated in CST Studio Suite. C represents the capacitance value of varactor D. The reflection spectrum at 4–6 GHz is simulated under the sweep of capacitances configuration. Figure 2e shows the simulated reflection amplitude curves of the meta-atom for different capacitances under the y-polarized incidence. As can be seen, the reflection amplitude value is very small and close to 0, indicating that adjusting the capacitance from 0.17 pF to 1.1 pF has no influence on the amplitude of the unit. Figure 2f illustrates the simulated phase curves. At 4.7–5.2 GHz, the reflection phase can roughly cover 360°, which can cope with the required phase compensation. The designed unit is suitable to realign the scattered wavefront.

**Figure 3:**
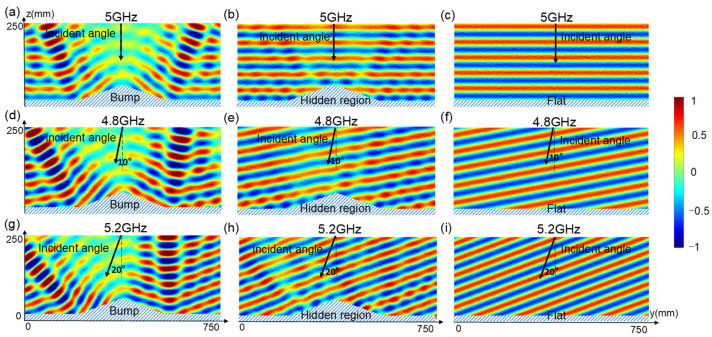
Full-wave simulations of an Ey-polarization metasurface cloak, indicating reflected transverse magnetic field distributions at 5 GHz when a plane wave is incident to a bare bump at normal incidence (**a**), a bump covered by a polarization-insensitive metasurface cloak (**b**), and a flat (**c**). Reflected transverse magnetic field distributions at 4.8 GHz when a plane wave is incident to a bare bump at an incidence angle of 10° (**d**), a bump covered by a polarization-insensitive metasurface cloak (**e**), and a flat (**f**). Reflected transverse magnetic field distributions at 5.2 GHz when a plane wave is incident to a bare bump at an incidence angle of 20° (**g**), a bump covered by a polarization-insensitive metasurface cloak (**h**), and a flat (**i**).

Then, full-wave simulations of a triangle metasurface cloak with a tilt angle of 18° were performed in CST Microwave Studio. In order to decrease computation in the simulation, the bevel is composed of 15 × 1 meta-atoms, that is, just one row of meta-atoms in the x direction. Metal planes extend at the ends of the cloak (Figure 3b,e,h). The wave is incident at y polarization. The periodic boundary condition was applied to both x sides and the open boundary condition was applied to other sides to ensure the simulation accuracy. Without the metasurface, a metal triangle block with the same size as the cloak, with metal planes extending, was simulated as a “bump” (Figure 3a,d,g), and a complete metal flat with the same projection size of the cloak and extending plane was simulated as “Flat” (Figure 3c,f,i). We simulated it under vertical incidence at 5 GHz, an oblique incidence angle of 10° at 4.8 GHz, and an oblique incidence angle of 20° at 5.2 GHz, respectively, setting corresponding capacitance values to verify the operative capacity in different incidence directions. When y-polarized waves normally impinge onto the bare bump, strong backward scattering is mainly divided into two directions, whose wavefront is obviously different from that generated by the flat metallic ground (Figure 3a,d,g). After wrapping the metasurface cloak, the local reflection characteristic of each meta-atom could be suitably modulated, leading to a distribution of the scattering field similar to the wavefront reflected from the metallic ground, as shown in Figure 3b,c. Figure 3e,f,h,i show that the cloak can also work well at the oblique incidence angle of 10° at 4.8 GHz and 20° at 5.2 GHz.

### 2.3. Direction Sensing Module and Feedback Control Module

**Figure 4:**
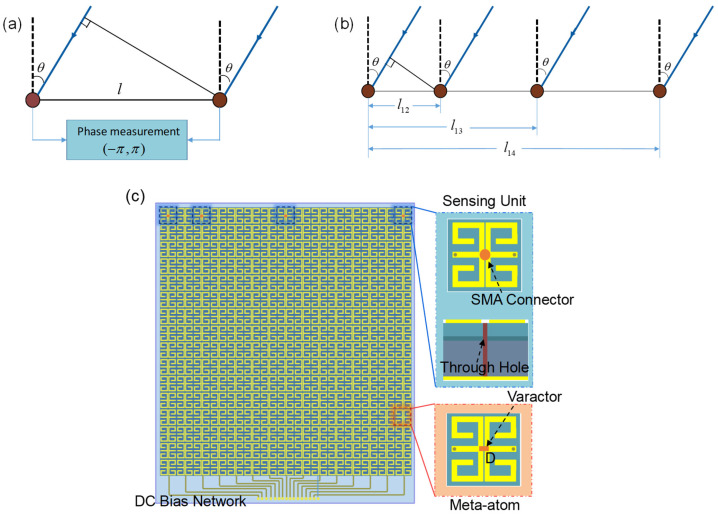
The principle and design of the sensing module. (**a**) Single-baseline phase interferometer. The phase difference between two points is related to the direction of the incident EM wave, but the phase measurement will be limited to (−π,π). (**b**) Designed phase interferometer system with four units. (**c**) Top view of the intelligent metasurface with sensing units and the bias network. The four sensing units receive the incident EM wave and are connected to the sensing module.

There are three main techniques used in passive direction finding, namely time difference of arrival, amplitude comparison, and phase interferometer. In order to protect the invisibility cloak from being affected, limited by the size of the metasurface and the computational power of the selected hardware, the phase interferometer is chosen. Figure 4a shows the principle. In far-field conditions, the incident wave can be regarded as a parallel wave. Due to the different spatial distribution of sensing units, when the incident wave is obliquely incident, the signal received by the left sensing unit is delayed and has a phase lag compared to the right one. When the distance between two units is l, the wavelength and angle of the incident wave is λ and θ. The phase lag Δϕ is:

(1) $$\Delta \phi =\frac{2\pi }{\lambda }\Delta d=\frac{2\pi }{\lambda }l\sin\theta$$

which is related to the direction of the incident wave. So, as long as the precise phase difference can be measured, the direction of incidence can be inferred, which is:

(2) $$\theta =\arcsin(\frac{\lambda \Delta \phi }{2\pi l})$$

generally speaking, the wider the antenna spacing, the higher the accuracy of the interferometer system. However, since the phase measurement moves only in the range of (−π, +π), the increment of antenna spacing leads to ambiguity in the angle of arrival (AOA) calculation when the antenna spacing is longer than half of the signal wavelength. So, the system usually uses one short baseline (<λ/2) and one or more larger baselines (>λ/2). The small baseline is set to obtain the unambiguous value, while the longer baselines help correct data and improve accuracy.

We configure four units, one short baseline and two longer baselines, as reflected in Figure 4b. The phase measurement value of the short baseline and the first long baseline are as follows:

(3) $$\begin{array}{l} \Delta \varphi_{1,2}=\frac{2\pi l_{12}}{\lambda }\sin\theta \\ \Delta \varphi_{1,3}+2p_{1}\pi =\frac{2\pi l_{13}}{\lambda }\sin\theta \end{array}$$

where $p_{1}=fix\{l_{13}\sin\theta /\lambda \}$, function fix(x) returns the integer part of the number x. Assuming the distance ratio between short baseline $l_{12}$ and long baseline $l_{13}$ is $l_{13}/l_{12}=m$, we can obtain:

(4) $$\Delta \varphi_{1,3}+2p_{1}\pi \approx m\Delta \varphi_{1,2}$$

and $p_{1}$ is:

(5) $$p_{1}\approx \frac{m\Delta \varphi_{1,2}-\Delta \varphi_{1,3}}{2\pi }$$

round the obtained p and substitute it into:

(6) $$\Delta \phi_{1,3}=\Delta \varphi_{1,3}+2p_{1}\pi$$

where $\Delta \phi_{1,3}$ is real phase difference in the long baseline. Further, regard $l_{13}$ as the short baseline and $l_{14}$ as the long baseline, $l_{14}/l_{13}=q$; we can obtain:

(7) $$p_{2}\approx \frac{q\Delta \varphi_{1,3}-\Delta \varphi_{1,4}}{2\pi }$$

so,

(8) $$\Delta \phi_{1,4}=\Delta \varphi_{1,4}+2p_{2}\pi$$

combined with Formula (2), we can obtain a relatively accurate direction angle.

Part of the meta-atoms are replaced with the above design. As shown in Figure 4c, the bevel of the invisibility cloak we process physically is composed of such a metasurface. There are 15 columns overall, each consisting of 16 meta-atoms and sharing the same bias voltage configuration in the 2D cases. The sensing unit is placed at the other end, away from the power header, and replaces the 1st, 3rd, 8th, and 15th meta-atoms from the left according to the distribution in Figure 4c. The length of two meta-atoms is almost exactly less than half the wavelength (the length of two units is 26 mm and λ > 57.7 mm when frequency ranges from 4.7 to 5.2 GHz), and the distance ratios between the second sensing unit and the third, as well as the third and the fourth, were selected as prime numbers based on the reference literature [34]. In order to be consistent with the other meta-atoms and simultaneously be able to absorb the energy of the incident wave, a sensing point (via a hole with a diameter of 1.2 mm) is designed to replace the varactor and solder with a sub miniature version A (SMA) connector, which is in the gap between two mental patterns. Thus, the energy of the spatial incidence captured by the two metallic inner patches in the sensing unit goes through the frequency sensing module via the SMA connector. Sensing units are connected to the direction sensing module, which is used for detecting the frequency and direction of the incident EM waves. We choose AD9361 and Zedboard to process the received signals. Down conversion and analog-to-digital conversion are performed in AD9361. Then, in Zedboard, the frequency and phase information are calculated by the FFT algorithm, and finally converted into a directional angle by phase interferometry.

Then, we use serial peripheral interface (SPI) communication to transmit the direction information to STM32f103 as a micro-controller unit (MCU). The MCU is the core of the adaptive feedback control module. It is used to calculate the required compensation phase for each column to present the metasurface cloak according to the frequency and direction information, find the corresponding voltage for controlling the varactor diode to obtain the compensation phase, and distribute all the commands to the varactor diodes through the programmable DC sources. Since each column of metasurface requires one independent bias line to drive the diodes, the control module needs 32 channels of DC signal supply in total. EVAL-AD5535 is a DAC that can output 32 channel voltages. So, it is suitable for controlling the voltage of the metasurface.

As indicated above, the overall system has formed a closed loop. The proposed intelligent cloak system can adaptively match the recognized frequency and direction of the incoming waves, and the cloak system can work in most cases by adjusting appropriate voltage configuration to realize the independent control of the reflection phase.

## 3. Experiment Results and Discussion

### 3.1. Reflection Spectrum Verification of the Meta-Atom and Relationship Measurement between the Reflected Phase and the Control Voltage

**Figure 5:**
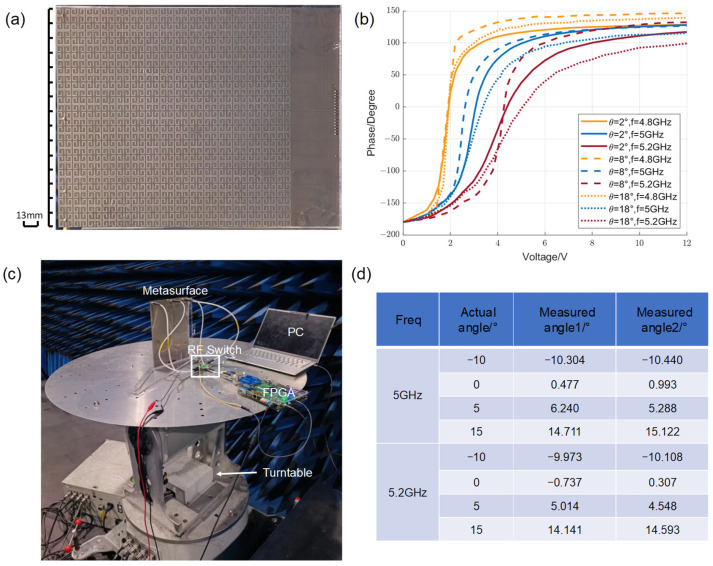
(**a**) Photographs of the metasurface sample. (**b**) The reflection phase response curves for changing bias voltages when the incident wave is incident at different incidence angles and frequencies, respectively. (**c**) The experimental setup of direction detection module testing. (**d**) The experimental results of direction detection module testing.

To further verify the reflection characteristics and direct relationship between the reflected phase of the unit and the control voltage, a sample was fabricated and measured (Figure 5a). The metasurface sample, composed of 15 × 16 units, was fabricated using printed circuit board technology. A total of 236 varactor diodes and 4 SMA connectors were machined-welded into the sample. The whole metasurface, containing 15 columns of units, is controlled at the same time with consistent bias voltage configurations.

The experiment was carried out in a microwave anechoic chamber. Two standard gain horn antennas operating in the 4–6 GHz frequency band were connected to the vector network analyzer as the emission source and receiving end, respectively, at a few meters away from the metasurface, adjusting the transmitting antenna to change the angle of incidence. The voltage was set from 0 V to 12 V depending on the physical property of the varactor diode. The S_21_ parameters in the VNA were varied by tuning the DC sources, which showed the curve of the reflection phase as a function of voltage (Figure 5b). The results were acquired after calibration the waveguide with DUT, replaced with copper of the same size. The curves show that the reflection phase can roughly cover 360° when the voltage changes from 0 V to 12 V at 4.8–5.2 GHz. However, the curve slope at 1–3 V at 4.8 GHz is too large, which will affect the cloaking effect, so it should be discarded to narrow down the experimental scope to 5–5.2 GHz.

### 3.2. Performance Testing of the Direction-Sensing Module

To test the performance of the direction-sensing module, we attached one piece of the metasurface sample to the designed sensing module. As there are four sensing units for one piece of metasurface but AD9361 only has two receiving channels, an RF switch is used to select the input of the last three units, ensuring that the phases of the two units that make up the baseline are input simultaneously. The output was temporarily connected to the PC via the serial port, displaying and comparing the accuracy of direction measurement. Placing the above equipment on a turntable (Figure 5c) with a horn antenna at a distance of 2 m, the measurement angle was compared with the setting angle of the turntable. Figure 5d lists the results. The measurement error is within 1.5°.

### 3.3. Experimental Realization of Intelligent Metasurface Cloak System with Self-Adaptive Direction Identification

**Figure 6:**
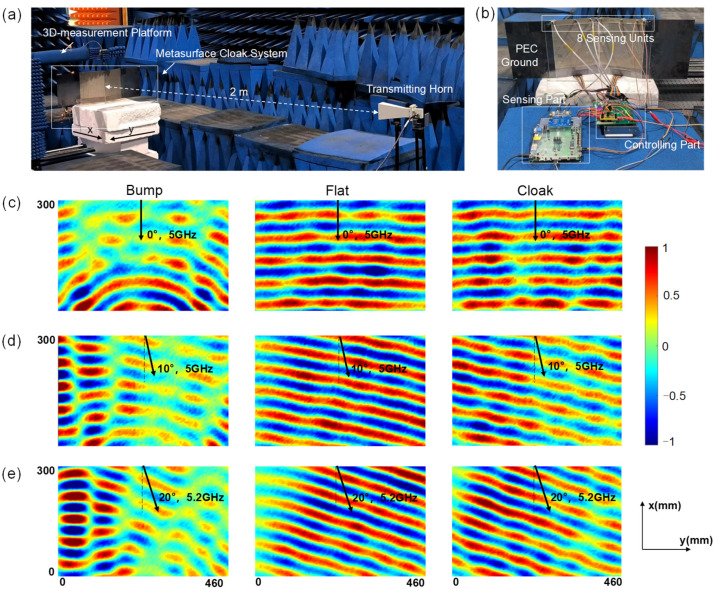
The experimental setup and results of intelligent metasurface cloak system with integrated sensing units. (**a**) The overall experimental setup. A TM-polarized beam from a transmitting horn is incident on the cloak system with incident angle θ. The observation region (460 mm × 300 mm) is at a height of 90 mm. The incident wave is detected in real time by the designed detection units, fed into the direction detection module to calculate the direction angle, sent to the feedback module, and all bias voltages are instantly calculated and supplied to the metasurface cloak. (**b**) The setup of the metasurface cloak system only, including the bump, PEC ground, metasurface, sensing part and controlling part. (**c**–**e**) Near-field reflected magnetic field Hx distributions of the bump, mental flat, and bump covered by the cloak when the wave is incident with an azimuthal angle of θ = 0° at 5 GHz (**c**), θ = 10° at 5 GHz (**d**), θ = 20° at 5.2 GHz (**e**). The black arrows represent the incidence direction of the EM wave.

To clearly explain the whole intelligent metasurface cloak system performance, an experimental realization was performed in a microwave anechoic chamber (Figure 6a). In the experiment, a metal bump was covered with two identical metasurfaces tilted at 18° and connected to metal plates to simulate placement on the PEC ground. The bump illustrates the capacity of the cloaking space, which is applicable to objects of any shape. The power supply lines of each column were connected to the feedback control module. Sensing units were connected to the sensing module by SMA connectors and appropriate RF switches (Figure 6b). A standard gain horn antenna operating in the 4–6 GHz frequency band was connected to one end of the vector network analyzer as the emission source, and the other end was connected to a measurement loop antenna, which was fixed on a mechanical arm of a 3D measurement platform controlled to move in the YZ plane to point-to-point probe the magnetic field. The electric field was polarized along the y direction. We fixed the entire cloaking device system to a turntable and placed the transmitting horn a few meters away.

During the experiment, the sensing units on the cloak received a signal from the transmitting antenna, and transmitted it to the sensing module. The sensing module refreshed data, updated the calculated directional information, and passed it to the feedback control module by SPI communication. Then, the control module calculated and changed the voltages applied to the varactor diodes on the units according to the direction data, causing the reflection phase to be amended. The observation of whether the measured electric field demonstrated the good performance of the cloak allows for the determination of whether the overall system worked successfully. In the event of a change in the incident wave frequency, it is necessary to actively press the button on the FPGA in the sensing module to update the sensing frequency.

The experiment only considers the azimuth. The incident wave direction θ = 0° is defined as the transmitter incident vertical to the PEC ground. In the first case, we measured the field distributions when the wave with Hx polarization is normally incident to the sample from the top (θ = 0°). The incident field (pre-measuring the field distribution in the same area without placing any objects) was subtracted from the measured magnetic field to obtain the reflected field. The reflected magnetic field distributions are shown in Figure 6c. The figures show that when the EM wave impinges on the bump, the reflected beams are distorted and scattered into different directions. After covering it with a metasurface cloak, the distortion of the reflected phase and polarization are reconstructed, and the split beams rejoin again. One can see the measured results are very similar to those measured for the ground plan.

In the second case, we kept all the setups the same as Case I, but the direction of the incident wave changed (θ = 10°). From the results for the bare bump, metasurface cloak, and flat ground plane (Figure 6d), one can see that the metasurface cloak can effectively cancel the scattering fields caused by the bare PEC bump.

In the next step, we measured the case when the wave is incident with an azimuthal angle of θ = 20° at 5.2 GHz. The results are shown in Figure 6e. Good cloaking performance has been demonstrated from the measured electric field distribution in all planes, which further confirms the effectiveness of our cloak.

## 4. Conclusions

In this paper, we propose an intelligent metasurface cloak system for multiple directions, which integrates real-time frequency and direction sensing with an adaptive feedback electronic control system. This system adjusts the reflected phase by accurately recognizing the direction of incoming waves. We have designed, fabricated, and tested a programmable reflective metasurface with independent phase control, capable of adjusting its reflected phase nearly 360° through the tuning of varactor diodes. An integrated direction sensing module was developed for real-time detection, using detecting points to replace meta-atoms for enhanced accuracy and convenience. The feedback control module translates directional information into corresponding voltage to adjust the phase, forming a closed-loop system. The experimental results demonstrate that our intelligent metasurface cloak system can completely restore the polarization, amplitude, and phase of light in response to various incident directions, mimicking the effect of light reflecting off a flat mirror, with high performance and no need for manual control or intervention. Compared to the previous invisibility cloak, our system is more integrated and easily portable, and is capable of handling complex EM environments, as shown in Table 1. The proposed concept embodies the direct integration of perception and cloaking on the metasurface, and lays the foundation for the composite application of the intelligent metasurface cloak.

## Figures and Tables

**Table 1:** A comparison of the performance with previous work.

Reference	Passive/Active	Adaptive	Sensing	Integrated
[18]	Passive	No	No	No
[31]	Active	Yes	No	No
[32]	Active	Yes	Yes	No
Our work	Active	Yes	Yes	Yes

## Data Availability

The original contributions presented in the study are included in the article, further inquiries can be directed to the corresponding authors.
